# Novel RNAi-Mediated Approach to G Protein-Coupled Receptor Deorphanization: Proof of Principle and Characterization of a Planarian 5-HT Receptor

**DOI:** 10.1371/journal.pone.0040787

**Published:** 2012-07-18

**Authors:** Mostafa Zamanian, Prince N. Agbedanu, Nicolas J. Wheeler, Paul McVeigh, Michael J. Kimber, Tim A. Day

**Affiliations:** 1 Neuroscience Program, Iowa State University, Ames, Iowa, United States of America; 2 Department of Biomedical Sciences, Iowa State University, Ames, Iowa, United States of America; 3 School of Biological Sciences, Queen’s University Belfast, Belfast United Kingdom; Wayne State University, United States of America

## Abstract

G protein-coupled receptors (GPCRs) represent the largest known superfamily of membrane proteins extending throughout the Metazoa. There exists ample motivation to elucidate the functional properties of GPCRs given their role in signal transduction and their prominence as drug targets. In many target organisms, these efforts are hampered by the unreliable nature of heterologous receptor expression platforms. We validate and describe an alternative loss-of-function approach for ascertaining the ligand and G protein coupling properties of GPCRs in their native cell membrane environment. Our efforts are focused on the phylum Platyhelminthes, given the heavy health burden exacted by pathogenic flatworms, as well as the role of free-living flatworms as model organisms for the study of developmental biology. RNA interference (RNAi) was used in conjunction with a biochemical endpoint assay to monitor cAMP modulation in response to the translational suppression of individual receptors. As proof of principle, this approach was used to confirm the neuropeptide GYIRFamide as the cognate ligand for the planarian neuropeptide receptor GtNPR-1, while revealing its endogenous coupling to G*α_i/o_*. The method was then extended to deorphanize a novel G*α_s_*-coupled planarian serotonin receptor, DtSER-1. A bioinformatics protocol guided the selection of receptor candidates mediating 5-HT-evoked responses. These results provide functional data on a neurotransmitter central to flatworm biology, while establishing the great potential of an RNAi-based deorphanization protocol. Future work can help optimize and adapt this protocol for higher-throughput platforms as well as other phyla.

## Introduction

G protein-coupled receptors (GPCRs) have been the subject of intense research scrutiny due to their central role in eukaryotic signal transduction and their exploitability as drug targets [1]–[3]. Once identified, GPCRs typically undergo deorphanization, the process of pairing orphan receptors with their cognate ligands. Current approaches to GPCR deorphanization have severe limitations and are inefficient for large-scale projects. The predominant approaches all require the transient or stable heterologous expression of GPCRs in a surrogate cell system and in most cases, this expression occurs in cells derived from other species and phyla [4]–[6]. This has introduced a significant bottleneck in the way of both the pharmacological and structural characterization of GPCRs [4], [7].

The complex regulatory processes that guide the correct folding and export of receptors to the cell membrane [8]–[11] are not necessarily highly-conserved across cell lineages. In the event that a GPCR is successfully expressed on the surface of a host cell, the receptor must operate in conjunction with a foreign complement of accessory and signaling proteins. Further, the structural and functional integrity of receptors can be altered by local membrane composition [12], [13]. The exact post-translational requirements for proper receptor expression and function can vary greatly among receptors, making the task of identifying a suitable heterologous system unique to each receptor and, ultimately, dependent on trial and error [4].

Although heterologous expression is not a theoretically challenging feat, individual targets routinely prove to be recalcitrant and consume inordinate effort. In view of these concerns, a simple receptor deorphanization method that could be applied in a native cell or membrane environment could side-step some of these limitations.

### Flatworm GPCRs

The phylum Platyhelminthes houses prominent human pathogens as well as tractable model organisms. Flatworm GPCRs represent lucrative anthelmintic targets, as evidenced by the biological activities of their putative ligands [14], [15] and the crucial biological functions of these receptors in other organisms [16], [17]. Signaling pathways associated with the GPCR superfamily have been identified as potential targets for life-cycle interruption of flatworm parasites [18], [19]. The recent availability of platyhelminth genomic data [20]–[22] has led to the accumulation of a wealth of receptor and ligand data. A comprehensive *in silico* protocol revealed over 117 *Schistosoma mansoni* and 460 *Schmidtea mediterranea* GPCRs, which were classified using phylogenetic, homology-based, and machine-learning approaches [23]. Bioinformatics and proteomics-based studies have similarly led to the expansion of the known set of putative GPCR ligands [24], [25].

The pharmacological characterization of orphan flatworm receptors is likely to generate valuable drug discovery leads, while enhancing our understanding of basic receptor biology in this important phylum. Reliance on heterologous expression platforms have hampered efforts to implement functional assays to identify receptor agonists. Only a handful of flatworm GPCRs have thus far been deorphanized, with receptors expressed in such divergent cellular environments as CHO [26], HEK293 [27], [28], COS7 [27], yeast [28], [29], and *Xenopus* oocyte cells [30]. We describe a relatively simple loss-of-function deorphanization approach that could be applied in a native cell or membrane environment. This alternative strategy could help catalyze a first-pass mapping of receptors and ligands in this and other phyla.

### Inversing the Paradigm: RNAi as a Deorphanization Tool

We validate an RNA interference (RNAi)-based method that allows receptors to undergo deorphanization without the need for full-length cloning and transport to a heterologous expression system. In principle, a collection of putative ligands are screened against membrane preparations to evaluate their effects on second-messengers downstream of GPCR activation. RNAi is then used to assay whether observed responses can be altered or abolished by the knockdown of individual receptors from the membrane preparations. A successful “hit” confirms expression of a given receptor, functionally pairs the receptor with a given ligand, and couples the receptor with a specific G protein signaling pathway. Bioinformatics approaches can be used to help identify receptors as putative targets for a particular ligand, or conversely, to narrow the list of potential ligands for a given receptor.

The primary biochemical endpoints of GPCR activation are typically assayed by recording agonist-evoked changes in cAMP (G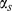 and G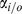) or Ca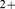 (G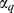) levels. A variety of established labeling and detection schemes (e.g. fluorescent, luminescent, and radioisotope) are available for these second messengers [31]. In this study, we focus our efforts on the G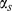 and G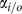 pathways and employ a radioimmunoassay (RIA) for cAMP detection. Monitoring adenylyl cyclase modulation of cAMP allows us to examine two of the three major GPCR activation endpoints.

**Figure 1:**
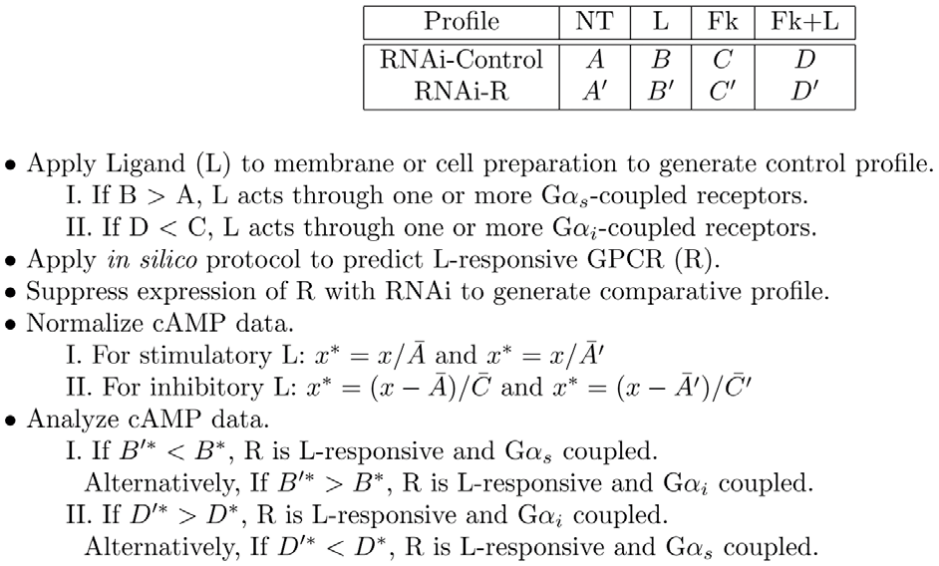
Logic of RNAi-based deorphanization experiment. The general set of experimental outcomes for an RNAi-based deorphanization experiment focused on the G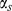 and G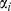 pathway are shown. Letters 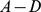 and 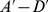 each represent cAMP datasets for particular treatment conditions. Potential results are described with respect to the notion that a given ligand may act on multiple GPCRs that are not necessarily coupled to the same G protein (G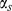 or G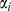). Abbreviations: NT, no treatment; Fk, forskolin; L, ligand; R, receptor; RNAi-control, control membrane preparation; RNAi-R, R-suppressed membrane preparation; 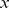, cAMP measurement variable. Asterisks (*) are used to denote normalized data.

While this loss-of-function approach limits pharmacological analysis, it is likely adaptable to higher-throughput platforms and can serve as an efficient ligand-receptor mapping tool for certain receptor classes. It should be noted that ligands and receptors can display pharmacological promiscuity; ligands can act through more than one receptor and receptors can respond to more than one ligand, with a range of affinities. Further, receptors responsive to a given ligand do not necessarily share the same G protein coupling profile and are likely to be expressed in different abundances. However, this approach only concerns itself with the contribution of individual receptors to differences between control and RNAi response profiles. The scale and directionality of these differences provide information relevant to ligand responsivity and G protein coupling, respectively. The basic logic of this deorphanization strategy is outlined in Figure 1.

## Results and Discussion

### cAMP Assay Optimization and Ligand Screen

A cell membrane preparation protocol was adapted [32] and optimized for planaria, and used to generate samples for treatment with putative GPCR ligands. The downstream effects of ligand incubation on cAMP levels were monitored using a cAMP RIA. A screen was first carried out on *Dugesia* (*Girardia*) *tigrina* membrane preparations with a small number of peptides and biogenic amines. These ligand classes are prominent in platyhelminth biology [14], [15], [24], [25], and there is a strong likelihood that a subset signals through one or more receptors coupled to either the G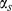 or G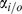 pathways. This would presumably be made apparent by stimulation of basal cAMP levels or inhibition of forskolin (Fk)-stimulated cAMP levels [33] as measured by RIA, respectively.

Included in this initial screen were the only two ligands definitively coupled to planarian GPCRs: the neuropeptide GYIRFamide and the biogenic amine serotonin (5-HT; 5-hydroxytryptamine). It was a reasonable assumption that both GYIRFamide and 5-HT would modulate cAMP levels in a whole organism membrane preparation. The *D. tigrina* receptor GtNPR-1 was previously deorphanized, showing a potent dose-dependent response to the neuropeptide GYIRFamide in mammalian cell culture [26]. Chimeric G proteins (G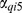 and G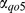) were used to divert downstream GtNPR-1 signaling through the G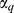 pathway, suggesting this receptor is G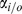-coupled in its native environment. More recently, a *Dugesia japonica* 5-HT GPCR has been deorphanized using *Xenopus laevis* oocytes [30], and there is long-established evidence of 5-HT stimulation of cAMP in both *S. mansoni* [34], [35] and other planarian species [36], suggesting that 5-HT acts through one or more G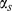-coupled GPCRs.

**Figure 2:**
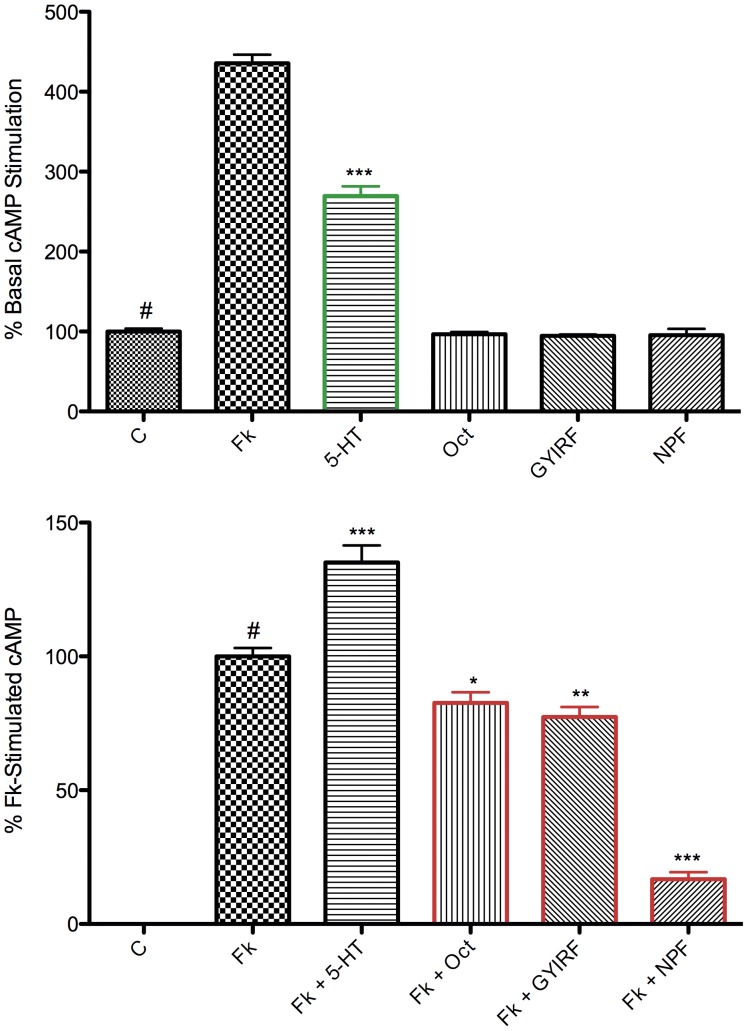
Peptide and biogenic amine ligand cAMP screen performed against isolated *D. tigrina* membranes. RIA cAMP outputs are normalized and shown as mean 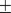 SEM, with asterisks representing statistically significant differences compared to a control bar (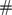); *P
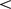
0.05, **P
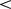
0.01, ***P
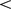
0.001, one-way ANOVA, Tukey *post hoc* test. Top: red-outlined bars signify ligands that stimulate cAMP compared to basal levels, likely mediated by G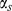-coupled GPCRs. Bottom: red-outlined bars signify ligand inhibition of Fk-stimulated cAMP, likely mediated by G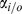-coupled GPCRs. Serotonin (5-HT) stimulates basal cAMP, while octopamine (Oct), GYRIFamide (GYIRF), and neuropeptide F (NPF) all inhibit Fk-stimulated cAMP at 100 uM. These changes in cAMP are presumably receptor-mediated, and should therefore be altered in a ligand-specific manner by subtraction of particular receptor targets from cell membranes via RNAi.

Alongside GYIRFamide and 5-HT, we included neuropeptide F (NPF) and octopamine as putative ligands. NPF has been shown to inhibit Fk-stimulated cAMP production in membranes isolated from *S. mansoni* [32]. Given the identification of planarian NPF homologues [24], [25], we hypothesized that this peptide would have a similar inhibitory effect on cAMP levels. The results of this primary screen show that 10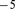 M 5-HT drastically stimulates cAMP production, with Fk and 5-HT together leading to greater cAMP production than either Fk or 5-HT alone (Figure 2a). No other putative ligand significantly increased cAMP compared to basal levels. Further, 10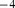 M GYIRFamide, 10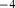 M NPF, and 10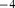 M octopamine inhibit Fk-stimulated cAMP accumulation in *Dugesia* membrane preparations (Figure 2b) to varying degrees. Inhibition was greatest for NPF at ∼82%, followed by GYIRFamide at ∼24%. These changes in [cAMP] can be viewed as the ‘additive response profile’ for each ligand. By this we refer only to the total measured effect of the ligand on the second messenger, as potentially mediated by one or more receptors. This is to acknowledge that different receptors that respond to the same ligand may contribute in different ways to the overall response being measured.

We chose to first pursue the response profiles of GYIRFamide, provided that GtNPR-1 is a known target of GYIRFamide in *D. tigrina*. As proof of principle, we investigated whether or not this would be apparent using this loss-of-function assay. Given that the inhibition of adenylate cyclase by GYIRFamide is less potent than that brought on by NPF, this also serves as a more difficult trial for validation of assay sensitivity.

### Coupling cAMP Assay with RNAi: GtNPR-1 Proof of Principle

#### Establishing RNAi-mediated receptor suppression

**Figure 3:**
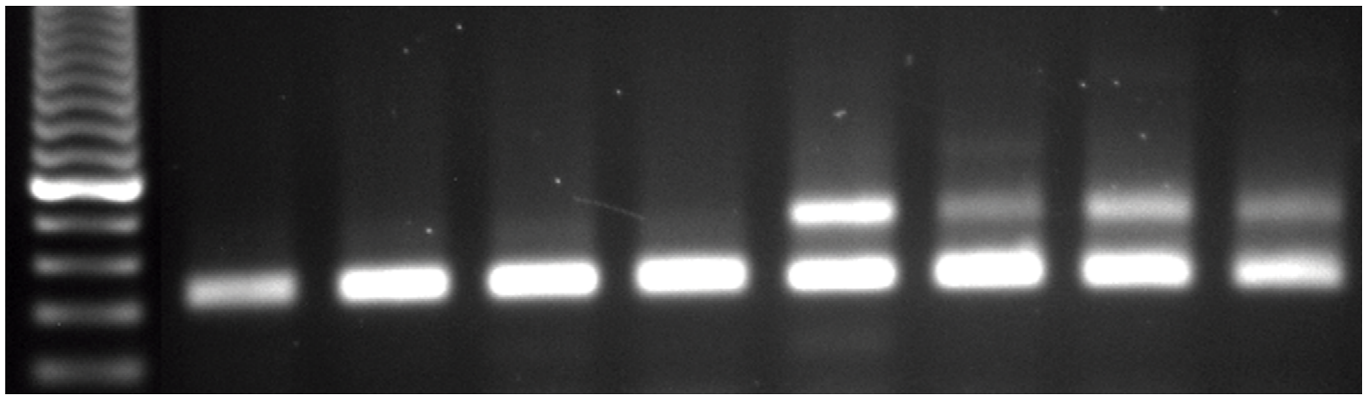
Semi-quantitative PCR reveals *GtNPR-1* knockdown. Lane 1 is a 100 bp DNA ladder, lanes 2–5 represent individual *GtNPR-1* dsRNA-fed planarians, and lanes 6–9 represent control dsRNA-fed planarians. The bottom band (∼300 bp) is the 18S internal standard, and the top band (∼400 bp) shows *GtNPR-1* expression. The top band disappears in the experimental group, confirming near abolishment of receptor expression in these worms. Relative band intensities (*GtNPR-1*/18S rRNA) for *GtNPR-1* RNAi group: 0.44
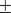
0.15. Relative band intensities for control group (band location manually selected): 0.08
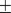
0.02. This corresponds to 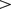
80% knockdown of *GtNPR-1* transcript.

Double-stranded (ds) RNA was introduced to isolated *D. tigrina* colonies using a bacterial-mediated feeding protocol. Planaria were randomly selected, isolated into treatment groups, and fed either non-flatworm control dsRNA or *GtNPR-1* dsRNA. A two-week RNAi feeding cycle consisted of four evenly-spaced feedings, followed by a four-day starvation period. Semi-quantitative RT-PCR was used to confirm mRNA knockdown. A small number of planarians were randomly selected from both experimental and control groups to assay *GtNPR-1* suppression, and the remaining planarians were used for membrane assays. Significant *GtNPR-1* knockdown (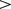 80%) is consistent and apparent in the experimental group, while *GtNPR-1* expression remains robust in the control group (Figure 3) relative to endogenous standard.

#### Deorphanization via comparison of response profiles

**Figure 4:**
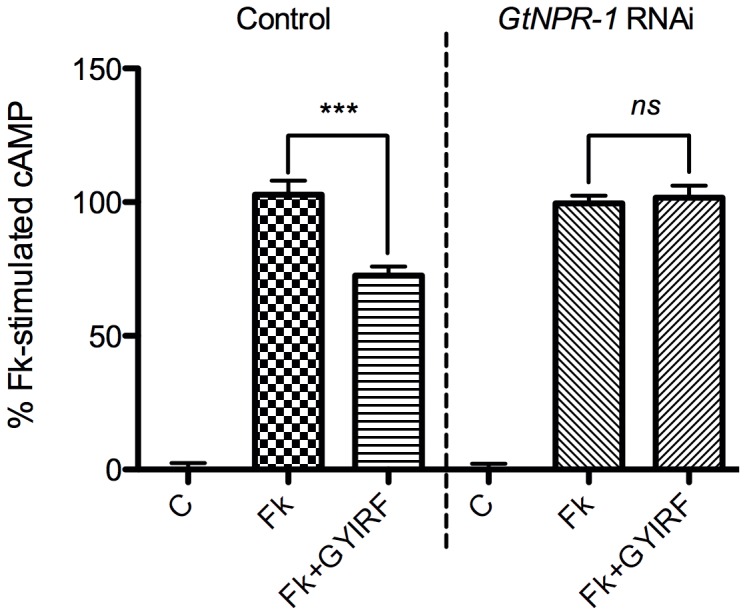
RNAi-based GtNPR-1 deorphanization. Treatment groups are Control (control dsRNA) and *GtNPR-1* RNAi (*GtNPR-1* dsRNA). Treatments are C (control), Fk (10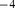 M forskolin), and Fk 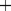 GYIRF (10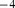 M forskolin and 10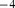 M GYIRFamide). Each bar is the mean (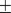 SEM) of 3 individual experiments. Basal cAMP levels were set as a baseline for each individual experiment, and cAMP values were normalized with respect to the level of Fk-stimulated cAMP (set at 100%). This allowed us to join datasets with differing basal cAMP levels, due to variance in the quality and yield of individual membrane preparations. Analysis of the raw cAMP values of individual experiments renders the same results (Table 1). Asterisks indicate significance at P
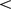
0.001 (***), and “ns” indicates no significant difference (one-way ANOVA, Tukey *post hoc* test).

Membranes were prepared from both control and *GtNPR-1* dsRNA-fed planarians, and treated with Fk (10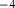 M), GYIRFamide (10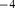 M), and Fk (10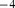 M)
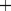
GYIRFamide (10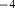 M). RIA was used to assay cAMP levels corresponding to these treatments. Comparison of the response profiles reveals near-complete abolishment of GYIRFamide-evoked inhibition of Fk-stimulated cAMP in the *GtNPR-1* knockdown group (Figure 4, Table 1). Overall, GYIRFamide reduces Fk-stimulated cAMP production by an average of ∼30% in the control group, and this inhibition was completely abolished by the suppression of *GtNPR-1* expression in the RNAi group. These results confirm that GtNPR-1 is agonized by GYIRFamide and further establish that this receptor is natively coupled to the G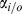 signaling pathway.

**Table 1:** RNAi-based GtNPR deorphanization cAMP raw values.

EXP	Treatment	Control	*GtNPR1* RNAi
	C	62.05±2.46	60.83±1.91
1	Fk	102.45±4.06	101.47±1.59
	Fk + G	85.02±1.59^***^	103.03±4.27*^ns^*
	C	27.88±0.97	33.54±1.27
2	Fk	57.37±2.68	58.78±1.64
	Fk + G	48.67±1.23^**^	57.89±0.93*^ns^*
	C	81.49±4.06	55.16±1.60
3	Fk	215.96±10.99	129.79±3.61
	Fk + G	195.63±6.17^**^	132.60±4.62*^ns^*

RIA-determined cAMP values (pM) are provided for three separate experiments (mean 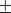 SEM). Treatments: C (control), Fk (Forskolin), Fk + G (Forskolin + GYIRFamide). The amount of isolated membrane differs between experiments, as evidenced by basal cAMP levels. This is in part due to differences in the size, number, and feeding behavior of worm batches used for membrane isolation. Analysis (one-way ANOVA, Tukey) of these raw datasets establishes abolishment of cAMP inhibition brought on by GYIRFamide associated with *GtNPR-1* suppression. For each experimental grouping, Fk is compared to Fk + G. Asterisks indicate significance at P
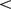
0.001 (***), P
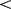
0.01 (**), and “ns” means no significant difference.

### *In silico* Target Selection

The two ligands that most drastically stimulated and inhibited adenlyate cyclase activity in our primary ligand screen were 5-HT and NPF, respectively. We decided to focus on 5-HT in an attempt to deorphanize a G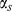-coupled receptor. To identify and rank 5-HT receptor candidates, a profile HMM was built with sequences procured from GPCRDB [37]. Training was focused on 62 full-length invertebrate 5-HT and 5-HT-like receptors. This model was used to search against *S. mediterranea* GPCR sequence datasets [23] and the results were ranked by E-value. The top 20 receptor candidates were used as BLASTp [38] queries against the NBCI “nr” database. This was used to identify receptors displaying 5-HT receptor homology, and to filter against receptors that displayed a non-specific range of biogenic amine receptor-related homology.

**Table 2:** 5-HT receptor candidate selection.

S. mediterranea	HMM	BLAST	TM	S. mansoni	D. japonica
mk4.013690.00.01	2.90E−108	+	6	Smp160020 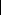 8E-45 (157)	
**mk4.005939.01.01**	**3.90E−85**	+	**6**	**Smp148210 | 8E-64 (195)**	**DjSER-7 | 3E-73 (364)**
mk4.011371.00.01	1.20E−71	−	7		
**mk4.001585.00.01**	**3.00E−70**	+	**5**	**Smp126730 | 9E-65 (258)**	**5HTLpla4 | 1E-141 (310)**
mk4.007388.02.01	9.60E−69	+	5		5HTLpla1 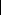 1E-127 (298)
mk4.029325.00.01	2.30E−65	−	7		
mk4.000656.10.01	1.10E−61	−	7		
mk4.004462.02.01	5.10E−51	+	5		DjSER-7 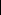 1E-115 (227)
mk4.011006.00.01	3.30E−50	+	5		
mk4.003202.01.01	1.10E−49	−	7		
mk4.000943.09.01	1.80E−48	+	5	Smp149770 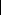 2E-31 (113)	
mk4.000354.16.01	1.70E−47	−	7		
mk4.012659.00.01	5.60E−46	−	7		
mk4.011160.01.01	2.90E−45	−	7		
mk4.000742.09.01	5.00E−44	−	7		
mk4.001678.03.01	1.20E−40	−	7		
mk4.010158.01.01	1.60E−38	+	5		
mk4.013827.00.01	7.80E−35	+	5	Smp126730 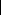 1E-59 (176)	
**mk4.001587.06.01**	**1.60E−29**	+	**5**	**Smp148210 | 8E-63 (179)**	**5HTLpla1 | 2E-63 (234)**
mk4.000526.00.01	3.60E−28	+	6		
mk4.017426.00.01	–	−	5		
mk4.017583.00.01	–	+	5		
mk4.012214.00.01	–	+	3		
mk4.011860.00.01	–	+	3		
mk4.012270.00.01	–	+	3		
mk4.013819.05.01	–	+	3	Smp149770 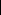 2E-34 (135)	
mk4.010946.00.01	–	+	5		

5-HT profile HMM hits are ranked by E-value for *S. mediterranea*. Additional sequences were appended via homology searches. This putative list of planarian 5-HT receptors was searched against the NCBI nr database using BLASTp. Receptors that exclusively showed serotonin-related homology in their top returned hits are marked with ‘+’. HMMTOP [50] was used to predict the number of TM domains for each sequence. Putative 5-HT receptors from *S. mansoni* [23] and *D. japonica* [51] were searched against the filtered HMM pool. The two nearest-related homologs for each of four *S. mansoni* receptors are shown, along with E-value and overlap length for each pairing. Similarly, the top pairings for each of three *D. japonica* receptors are shown. Three sequence clusters (bold) show high sequence conservation between parasite and planarian sequences. DjSER-7 has been previously deorphanized [30] and we therefore excluded this cluster from further consideration. Among the two remaining options, our choice of the highlighted sequence cluster is justified as follows: 1) the planarian sequences in this grouping share the highest level of sequence identity with their parasite sequelog, 2) the presence of two closely-related planarian sequences improves the likelihood of success for degenerate PCR as a strategy to amplify the *D. tigrina* homolog, and 3) deorphanization of a receptor in this cluster will assign a pharmacological identity to a novel subset of GPCRs.

**Figure 5:**
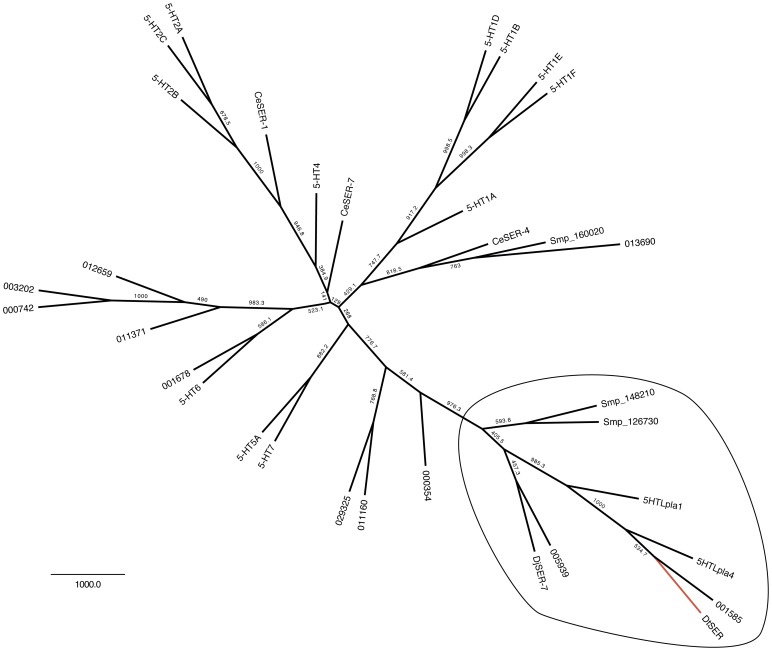
Maximum parsimony tree of serotonin receptors. Phylogenetic analysis was performed using planarian (*S. mediterranea* and *D. japonica*), parasite (*S. mansoni*), human and *C. elegans* 5-HT receptors and putative 5-HT receptors. TM domains I-VII were were extracted from the alignment for bootstrapping (bootstrap value 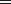 1000). Outlined receptors are significantly diverged from vertebrate and ecdysozoan serotonin receptors. DtSER-1 (red) was amplified using a degenerate PCR strategy and was chosen to undergo RNAi-based deorphanization.

**Figure 6:**
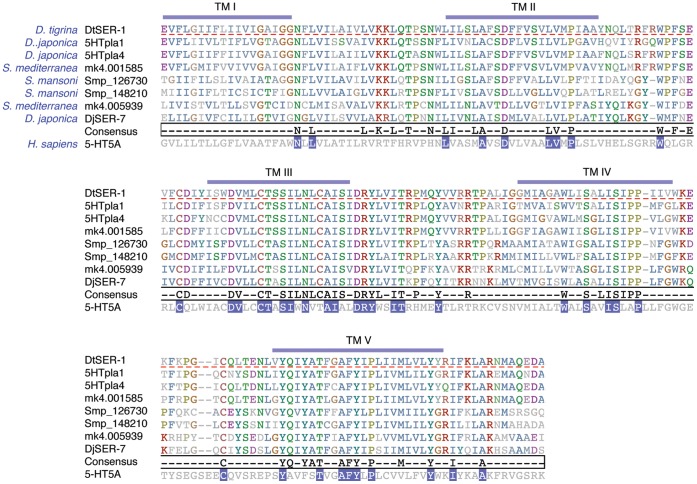
Multiple sequence alignment of serotonin receptors. DtSER-1 is shown aligned with other putative flatworm 5-HT receptor sequences that fall into to the same phylogenetic grouping (highlighted in Figure 5). This analysis encompasses low-entropy TM domains I–V, and TM domains are demarcated above the alignment in blue as predicted by HMMTOP [50]. Consensus (absolutely conserved) residues are shown for the flatworm receptors, and those conserved between this flatworm receptor grouping and a human serotonin receptor (5-HT5A) are highlighted in blue.

Receptors that survived this filter were compared to their nearest-related *S. mansoni* and *D. japonica* homologs (Table 2). While the bioinformatics evidence suggests multiple receptor targets for 5-HT, we narrowed our list to the best-conserved receptors between parasitic and free-living flatworms and used degenerate PCR to amplify a putative 5-HT receptor from *D. tigrina*. The selection strategy is outlined in Table 2. The amplified receptor is labeled DtSER-1 and maximum parsimony phylogenetic analysis places this receptor among a group of putative free-living and parasitic flatworm 5-HT receptors that are significantly diverged from those found in other phyla (Figure 5, Figure 6).

### RNAi-based Deorphanization of Planarian 5-HT Receptor

**Figure 7:**
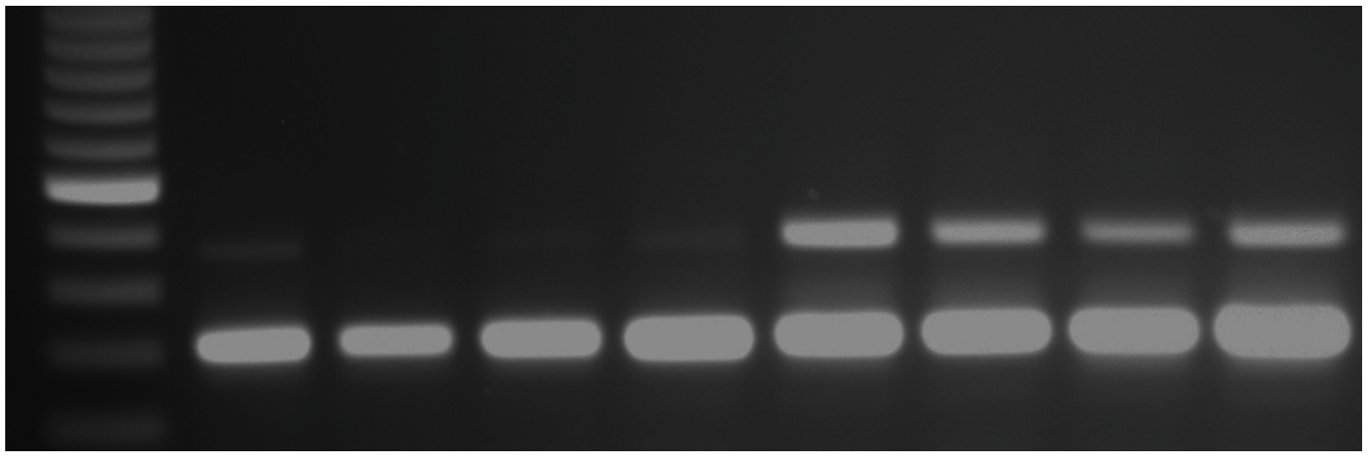
Semi-quantitative PCR reveals *DtSER-1* knockdown. Lane 1 is a 100 bp DNA ladder, lanes 2–5 represent individual *DtSER-1* dsRNA-fed planarians, and lanes 6–9 represent control dsRNA-fed planarians. The bottom band (∼300 bp) is the 18S internal standard, and the top band (∼480 bp) shows *DtSER-1* expression. The top band disappears in the experimental group, confirming near abolishment of *DtSER-1* receptor expression in these worms.

**Figure 8:**
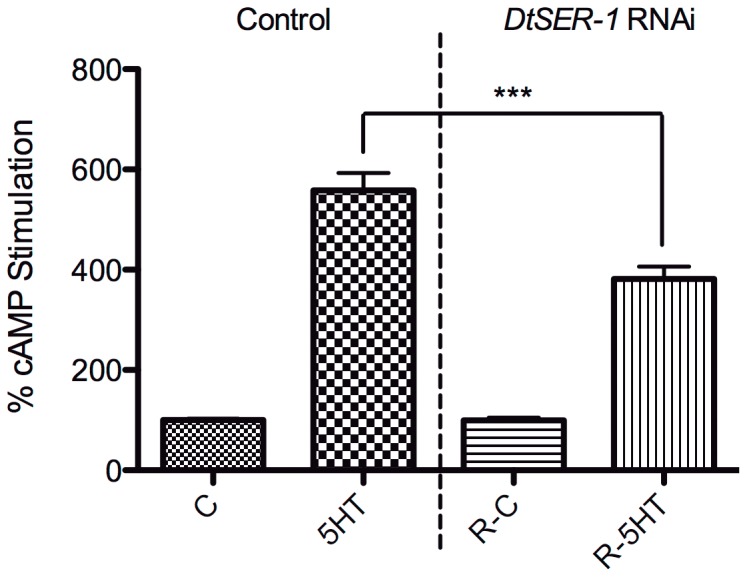
RNAi-based DtSER-1 deorphanization. Treatment groups are Control (control dsRNA) and *DtSER-1* RNAi (*DtSER-1* dsRNA). Treatments are C (control) and 5-HT (10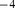 M). Each bar is the mean (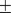 SEM) of 3 individual experiments. cAMP levels were normalized to basal cAMP levels (set at 100%), and the datasets were joined. *DtSER-1* knockdown corresponds to significantly decreased cAMP stimulation (∼32%) in response to 5-HT. Analysis of the raw cAMP values of individual experiments renders the same results (Table 3). Asterisks indicate significance at P
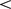
0.001 (***), and “ns” indicates no significant difference (one-way ANOVA, Tukey *post hoc* test).

DtSER-1 transcript expression was confirmed via PCR, and knockdown was elicited following the protocol described for GtNPR-1. Similar levels of transcript knockdown were obtained (Figure 7). Membranes from control and *DtSER-1* dsRNA-fed worms were isolated and treated with 5-HT (10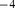 M). The response profiles reveal a significant decrease (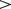
30%) in 5-HT evoked cAMP stimulation in the *DtSER-1* RNAi preparations compared to the control preparations. As with the neuropeptide receptor knockdown experiments, basal cAMP levels did not differ between control and experimental groups (Figure 8, Table 3). These results signify the successful deorphanization of DtSER-1 in its native membrane environment. DtSER-1 responds to 5-HT and is coupled to the G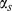 pathway. Serotonin receptors are implicated in motility and regeneration due to the phenotypic effects of serotonin in this phylum [39], [40]. Given that this receptor mediates significant increases in cAMP levels in response to serotonin, it is likely involved in these or other important physiological processes.

**Table 3:** RNAi-based DtSER-1 deorphanization cAMP raw values.

EXP	Treatment	Control	*DtSER-1* RNAi	Sig	Δ cAMP stimulation
1	C	29.43±1.26	30.43±2.56	ns	
	5-HT	153.29±4.89	99.18±2.26	***	−37%
2	C	129.33±4.57	114.29±13.22	ns	
	5-HT	498.56±39.86	324.32±42.00	**	−26%
3	C	14.71±0.91	18.02±1.040	ns	
	5-HT	113.18±4.42	96.42±1.69	***	−31%

RIA-determined cAMP values (pM) are provided for three separate experiments (mean 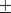 SEM). Treatments: C (control) and 5-HT (serotonin). Analysis (one-way ANOVA, Tukey) of these raw datasets establishes a significant decrease in 5-HT mediated cAMP stimulation associated with *DtSER-1* suppression. For each experiment, Control groups and 5-HT treated groups are compared between Control RNAi and *DtSER-1* RNAi conditions. Asterisks indicate significance at P
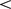
0.001 (***), P
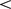
0.01 (**), and “ns” means no significant difference.

### Conclusions

This study establishes the utility of combining RNAi with biochemical endpoint assays as a means of deorphanizing GPCRs in their native membrane environment. The approach was first validated using the only deorphanized flatworm neuropeptide GPCR (GtNPR-1), confirming agonism by GYIRFamide while providing information about its endogenous G protein coupling profile. The orphan *D. tigrina* GPCR DtSER-1 was shown to respond to 5-HT, revealing its endogenous G protein pathway and illustrating the utility of applying an *in silico* strategy to candidate receptor selection. While this loss-of-function strategy side-steps some of the concerns and difficulties associated with heterologous GPCR expression, there is significant room for improving both the sensitivity and scalability of this assay.

The heavy tissue requirements of the membrane preparation protocols employed introduce a potential rate-limiting step. Further optimizations of membrane or whole cell preparation protocols in this phylum could allow for more efficient and robust pharmacological analysis. This assay could conceivably be adapted to higher-throughput platforms, extended to include GPCRs that signal through the G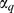 pathway, and employed in other phyla that are amenable to RNA interference-mediated gene knockdown. Conveniently, establishing receptor-specific RNAi in planaria allows for the accumulation of loss-of-function phenotypic data in parallel to pharmacological data. In this regard, the study of planarians can inform flatworm parasite biology. Biasing the receptor and ligand pool to those best conserved between parasitic and free-living flatworms could shed light on new targets for chemotherapeutic intervention.

## Materials and Methods

### Planarian Maintenance

*Dugesia tigrina* (Ward’s Natural Science, Rochester, NY) colonies were maintained in the laboratory in aerated spring water on a regular feeding cycle (∼2–3 times per week). Planaria were randomly selected and isolated in ∼50-worm groupings for RNAi feeding cycles and cAMP assays.

### RNA Interference

Primer3 [41] was used to select primers to selectively amplify 400–600 bp fragments of *GtNPR-1* and 5HT receptor candidate *DtSER-1*. BLAT [42] was used to help guard against potential off-target effects of silencing triggers using the very nearly-related *S. mediterranea* genome. A 465 bp fragment of *GtNPR-1* was amplified from a full length clone of *Gt-NPR1* housed in pcDNA3.1(+), with the primers 5′-TGGATCTTTCCAGCGACTCT-3′ (forward) and 5′-ATGGTTCGTTCGACGTTTTC-3′ (reverse). A 586 bp fragment of *DtSER-1* was amplified from *D. tigrina* cDNA isolated using RNAqueous (Ambion) and RETRoscript (Ambion), with a degenerate forward primer: 5′-GGKATGGAAGTATTTCTGGGRAT-3′ (forward) and 5′-TGGCATCTTCTTG GGCCATATTTCT-3′ (reverse). An RNAi control sequence was amplified from *Aedis aegypti* cDNA with primers 5′-AATGCCGGCCTGTTTCCTAT-3′ (forward) and 5′-AGCATCCTTTTTCTTGTGCG-3′ (reverse), corresponding to a putative odorant receptor (VectorBase id: AAEL013422 [43]). Second-round PCR was performed for each target sequence using the original gene-specific primers flanked by Gateway Cloning system (Invitrogen) recombination sites: 5′-GGGG- *attB1*-3′ (forward) and 5′-GGGG- *attB2*-3′ (reverse). Entry sequences were subcloned into the pPR244 (pDONRdT7) [44] destination vector with corresponding *attP1* and *attP2* recombination sites using BP Clonase II (Invitrogen). Clones were transformed into TOP10 Electrocompetent *E. coli* (Invitrogen) and sequence confirmed. RNAi vectors were introduced to HT115(DE3) cells for transcription of dsRNA, followed by bacterial-mediated feeding per standard protocol [45].

### Semi-quantitative RT-PCR

Total RNA was extracted from individual *D. tigrina* with RNAqueous (Ambion), followed by removal of DNA contaminants with TURBO DNase (Ambion). First strand cDNA synthesis was carried out with the RETROscript kit (Ambion), as part of a two-stage RT-PCR. PCR optimization was carried out with the QuantumRNA 18S Internal Standards kit (Ambion) per manufacturer instructions. 18S ribosomal RNA was used as an endogenous standard for normalizing measures of gene expression and reducing sample-to-sample variation. cDNA samples were used in parallel as templates for multiplex PCR with gene-specific and 18S rRNA primer pairs. PCR reaction products were visualized on 1.2% electrophoretic gel with the Kodak Gel Logic 112 imaging system, and amplicon intensities were analyzed with standard software to derive relative transcript abundances.

### Membrane Preparation

Planaria were washed twice with cold cAMP buffer containing 50 mM sucrose, 50 mM glycylglycine, 10 mM creatine phosphate, 2 mM MgCl_2_, 0.5 mM isobutylmethylxanthine (IBMX), 1 mM dithiothreitol (DTT), 0.02 mM EGTA, 10 units/ml creatine kinase, and 0.01% bovine serum albumin. Worms were kept on ice for 5 min and then homogenized on ice for 2 min with a Teflon homogenizer. This preparation was centrifuged at 5,000×g for 5 min, with the pellet that included cell debris discarded. This centrifugation step was then repeated. The supernatant was centrifuged at 40,000×g for 30 min at 4°C. The supernatant was discarded, and the membrane-containing pellet was resuspended via sonication in cAMP buffer suplemented with 0.1 mM ATP and 0.1 mM GTP. Total suspension volume was set at 500 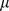
L/sample, such that each sample would contain cell membranes from ∼3 worms. 500 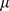
L aliquots of this membrane preparation correspond to individual reactions in the cAMP assay.

Samples were incubated with various concentrations (and combinations) of forskolin and/or putative ligands (peptide or biogenic amine) at 37°C for 20 min to stimulate cAMP production. Forskolin and peptide ligands were dissolved in DMSO, with final reaction mixtures containing 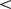
0.1% DMSO. DMSO has no measurable effect on cAMP in this range (data not shown). Samples were centrifuged at 3,000×g for 5 minutes after ligand incubation, and 400 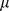
L of supernatant from each sample (3 samples per treatment) was transferred into a fresh tube for cAMP determination using radioimmunoassay.

### cAMP Determination

cAMP levels were measured with RIA as previously described [46] with minor modifications. 100 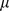
L aliquots from each sample or known standard (standard curve range: 4 – 512 fmol cAMP) were acetylated and incubated overnight at 4°C with primary cAMP antibody (1∶30,000) and cAMP[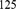
I] (∼20,000 cpm). 100 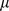
L of NRP (1∶80,000) and secondary antibody (goat anti-rabbit IgG; 1∶40,000) were added, followed by incubation at 25°C for 10 min. 100 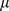
L of 50% normal bovine plasma and 1 mL of ice-cold PEG were added to the scintillation vials. Samples were centrifuged at 3,000 rpm (4°C) for 20 min. The supernatants were aspirated and 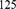
I levels in the pellets were assayed via gamma counter (Packard, B5002). For a given experiment, each sample was assayed in triplicate.

### Bioinformatics

HMMER-2.3.2 [47] was used to build a profile HMM for invertebrate 5-HT receptors. Training sequences were procured from GPCRDB and aligned with Muscle 3.6 [48]. The profile HMM was constructed with *hmmbuild* and calibrated with *hmmcalibrate*. This model was used to search a curated dataset of putative *S. mediterranea* receptors with *hmmpfam*. The resulting matches were ranked by E-value and the top 20 full-length hits were further examined. Putative hits were matched with their nearest-related *S. mansoni* and *D. japonica* homologs, and also searched against the NCBI nr database with BLASTp. Maximum parsimony phylogenetic analysis was carried out with the Phylip 3.6 [49] package.

### Statistical Analysis

In cases where a ligand had an overall inhibitory effect on Fk-stimulated cAMP, basal cAMP levels were set as a baseline for individual RIA experiments and cAMP values were normalized with respect to the level of Fk-stimulated cAMP (set at 100%). In cases where a ligand had an overall stimulatory effect on cAMP, cAMP values were normalized with respect to basal cAMP (set at 100%). This allowed us to join datasets from repeated experiments with differing basal cAMP levels due to variance in the quality and yield of individual membrane preparations. One-way analysis of variance (ANOVA) was used with Tukey’s *post hoc* test for multiple comparison analysis of cAMP levels associated with different treatments, for both normalized and raw values. Significances are reported at P
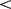
0.05, P
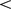
0.01, and P
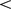
0.001.
